# Do middle-aged patients with onset of idiopathic scoliosis before the age of 10 years who have reduced pulmonary function have a risk for rapid decline – a comparative study

**DOI:** 10.1186/s12890-024-03053-2

**Published:** 2024-05-24

**Authors:** Aina J Danielsson, Kerstin Löfdahl Hällerman

**Affiliations:** 1 Department of Orthopedics, Institute of Clinical Sciences, Sahlgrenska Academy, University of Gothenburg, Gothenburg, Sweden; 2 Departments of Orthopedics, Sahlgrenska University Hospital, Gothenburg, Sweden; 3 Department of Respiratory Medicine, Department of Internal Medicine and Clinical Nutrition, Institute of Medicine, Sahlgrenska Academy, University of Gothenburg, Gothenburg, Sweden; 4 Department of Respiratory Medicine, Sahlgrenska University Hospital, Gothenburg, Sweden

**Keywords:** Scoliosis, Outcome, Long-term, Pulmonary function

## Abstract

**Back ground:**

Knowledge concerning pulmonary function in adult patients with onset of idiopathic scoliosis before age 10 is sparse. A long-term follow-up (FU, mean 26 years, > 12 years after treatment) of pulmonary function (PF) in patients treated with brace or surgery due to idiopathic scoliosis with onset before the age of 10 was earlier performed. To evaluate whether a more severe reduction in pulmonary function leads to more rapid deterioration within a four-year period, this study was performed.

**Methods:**

Twenty patients with the most reduced pulmonary function and 19 out of those with normal PF found at the long-term FU were reexamined 4 years later to evaluate further changes in pulmonary function. Patients underwent spirometry and arterial blood gas analysis and answered pulmonary symptom questionnaires.

**Results:**

70% of the reduced pulmonary function group had undergone surgery vs. 26% of the normal group. The mean age (47 vs. 43 years) at this FU and curve size (37° vs. 35°) at the 26-year FU were similar. The decline in forced vital capacity (FVC) % of predicted was similar in both groups over the four-year period, from 67 to 65% in the reduced PF group vs. 96 to 94% in the normal PF group. The total lung capacity (TLC) % of predicted did not change over time in either group. No patient reported worsening dyspnea symptoms. Only one patient in the reduced PF group showed low arterial oxygen tension, 8.4 kPa, not signifying respiratory insufficiency.

**Conclusion:**

The age-related decline in FVC and TLC % of predicted did not differ between those with reduced and those with normal pulmonary function at the 26-year follow-up. Thus, these data do not infer increased rate of decline in the most deteriorated patients.

## Background

In our earlier study patients with idiopathic scoliosis who were orthopedically untreated, were found with an increased risk for respiratory failure and death in patients with onset before the age of ten years [1]. Other studies have shown similar results [2, 3]. In our study it was evident that the most severe increase in mortality occurred around the age of 40 [1]. We also found in another study that patients with a vital capacity below 50% of predicted value had an increased risk for respiratory failure [4]. These patients showed deteriorated blood gases in middle-age.

There is a lack of knowledge regarding whether patients with orthopedically treated idiopathic scoliosis and onset before ten years run an increased risk for respiratory failure. We have recently presented pulmonary function data from a long-term (26 years) follow-up of surgically or brace-treated patients with onset of idiopathic scoliosis before the age of ten years [5]. Most of the patients, who now were in their middle-ages, had pulmonary function that was preserved more than 20 years after treatment even if the mean values of the whole group were in the lower normal range. We found a reduced vital capacity (less than 70% of normal values) in 19 out of the 124 investigated patients, but only five of those patients had a vital capacity less than 50% of the normal value. The rapidness of deterioration of pulmonary function in patients with reduced pulmonary function is currently unknown. Our question therefore is whether these patients are at risk for a rapid decline in pulmonary function and ultimately the development of respiratory failure.

To evaluate the risk for developing respiratory failure, we reexamined the patients with the lowest pulmonary function four years after the long-term follow-up with a repeated pulmonary function test and blood gas measurements. Patients with the best pulmonary function from the same long-term follow-up were used as a control group.

## Methods

### Patients

The patients in the present study were selected from a previously performed follow-up at a mean of 26 years after treatment (“26 Y long-term FU”), which included 124 consecutive patients with idiopathic scoliosis with onset before the age of ten years [5]. The mean age at the FU was 41 years, and all patients had at least 12 years after completed treatment. Information collected during this follow-up could be reused in this study.

All patients had been treated before skeletal maturity and treatment was performed between 1966 and 1992. Patients with medium sized curves (24–45 degrees) had undergone brace treatment with 22–24 h daily wearing until skeletal maturity (*n* = 68) if sufficient residual growth was found. Patients with larger curves (above 45–50 degrees) or with curve progression despite bracing (*n* = 56) were operated with distraction and fusion of the scoliotic part of the spine. The treatment strategy has previously been described [5] and is presented in Table 1.

**Table 1:** Description of treatment strategies used for individuals with idiopathic scoliosis in the study group

Curve sizes between 24 and 45 degrees				Curve sizes above 45 degrees (thoracic, thoracolumbar, or double primary curves) and above 50 degrees (lumbar curves)	
Start of treatment	Treatment methods	Length of treatment period	If insufficient effect of bracing or curve progression despite bracing	Start of treatment	Treatment methods
In case of curve progression and depending on residual growth	Milwaukee brace until 1974 and thereafter a Boston brace. The brace was worn for 22–24 h daily	Until skeletal maturity, defined as Risser 4 and a skeletal age of 16 years for girls and 18 for boys	Surgery	if significant growth left	Initially braced in order to postpone surgery. Surgery was performed before maturity (see below)
				If **not** significant growth left	Operated on with distraction and fusion, until 1995 performed by use of the Harrington instrumentation and thereafter with a system coupled and contoured for derotation

The authors were unbiased, i.e. they had not taken part in the original treatment of the patients.

### Study protocol

Twenty patients who had had the most reduced pulmonary function (R) and 19 patients who had normal pulmonary function (N) at the long-term FU performed at a mean of 26 years after completed treatment (“26 Y long-term FU”) were reexamined four years later (“30 Y long-term FU”) with spirometric measurements and TLC measurements. The intention was to compare results between the two groups and use the patients with the best, i.e. normal (N) pulmonary function as a control group. The twenty patients with the most reduced pulmonary function (R) were also investigated with arterial blood gas measurements performed in resting sitting position.

Patients underwent the same procedure on both occasions, a spirometry with evaluation of the pulmonary function by measurement of the Forced Vital Capacity (FVC), Forced Expiratory Volume in one second (FEV1) and Total Lung Capacity (TLC) and with calculation of the dyspnea score according to the MRC questionnaire.

### The scoliotic spine

Full standing PA and lateral digital roentgenograms of the spine were obtained at the 26 Y long-term FU, while previous radiographic examinations regarding pre- and posttreatment were scanned. The curve size was measured digitally by the first author (A.D.) using the Cobb method [6], and the level of the apical vertebra was noted. In patients with double primary curves, the upper curve was used in the analyses. Those measurements of curve size were also used for this study.

### Pulmonary function

Spirometric values for FVC and FEV1 were measured with a pressure-difference membrane method (Jaeger ®Masterscope) in both investigations. The ratio of FEV1/FVC was calculated to evaluate airway obstruction. All the values are presented as percent of the predicted normal value related to age, height and gender according to Quanjer 2012 [7]. The spirometric values were corrected for loss of height due to scoliosis according to Lindh [8].

Total lung capacity (TLC) values are presented in liters and in the percentage of predicted normal values according to Quanjer 1993 [9]. In all measurements, the body plethysmograph was used.

In the present follow-up, arterial blood gas measurement was performed. This included oxygen tension (PaO_2_), carbon dioxide tension (PaCO_2_) and standard bicarbonate, which were determined in samples of blood obtained from the patients in the sitting position after 10 min of rest. Respiratory failure was defined as PaO_2_ below 8 kPa and/or PaCO_2_ above 6,5 kPa, or both. The lower limit for normal oxygen tension is 10 kPa. The standard bicarbonate (SBC) normal range is 22–27 mmol/L.

All equipment was calibrated according to standard norms. Patients were investigated in the sitting position.

### Questionnaires on respiratory symptoms and smoking habits

For evaluations of dyspnea, wheezing and smoking habits, the translation of a British questionnaire (MRC) [10–12] was used, as in previous studies of patients with scoliosis [1, 13]. Dyspnea severity was graded with an increase in symptoms from 1 to 5. The “smoking habit” was the cigarette consumption/day that was or had ever been used by the patient, recalculated as the sum of the number of packages smoked per year (count of 20 cigarettes/package*).*

### Statistical methods

The results regarding continuous variables as well as changes in continuous variables are described with n, mean, SD, and range for each group. Medians were calculated but were not different from the means and are not presented. The results for categorical variables, including dichotomous variables, are described with numbers and percentages. For comparison of continuous variables between two groups, the Mann-Whitney U-test was used, and for comparison of dichotomous variables between two groups, Fisher’s exact test was used. All tests were two-tailed and conducted at the 5% significance level. The data were analyzed using version 9 of the SAS System for Windows.

The Human Research Ethics Committee at the Medical Faculty at Gothenburg University, Gothenburg, Sweden, approved the study # 081/07. All participants in the study gave written informed consent.

## Results

Table 2 displays basic information on the study groups.

**Table 2:** Characteristics of the current study group with repeat spirometry four years after the long-term follow-up

Mean, (SD) / (range) or *n* (%)	Total (*n* = 39)	NORMAL PF (*n* = 19)	REDUCED PF (*n* = 20)	*p*-value Normal vs. reduced
**Gender, female**	28 (71.8%)	15 (78.9%)	13 (65.0%)	ns (0.54)
**Treatment** group w. early onset of IS				
Brace treatment	20 (51.3%)	14 (73.7%)	6 (30.0%)	*p* = 0.015
Surgery	19 (48.7%)	5 (26.3%)	14 (70.0%)	
**Age at**				
Diagnosis^a^	6.2 (2.8) (0.1–9.5)	7.2 (2.1) (0.2–9.5)	5.2 (3.0) (0.1–9.3)	*p* = 0.018
First long-term FU (”26-Y FU”)	41.0 (6.9) (28.3–53.4)	39.2 (6.2) (28.6–53.4)	42.8 (7.3) (28.3–52.7)	ns (0.13)
Second long-term FU (”30-Y FU”)	45.2 (6.8) (32.5–57.5)	43.4 (6.2) (32.5–56.7)	46.9 (7.1) (32.8–57.5)	ns (0.11)
**Follow-up period from maturity/surgery**, Y				
Until the 26-Y long-term FU	26.0 (7.2) (12.1–40.1)	23.4 (5.7) (12.1–32.7)	28.4 (7.8) (12.5–40.1)	*p* = 0.032
Between the 26- and 30-Y long-term FU	4.1 (0.7) (2.4–5.3)	4.1 (0.8) (2.4–5.3)	4.1 (0.7) (3.0-5.1)	ns (0.26)
**Curve size (Cobb°, major curve) at**				
Start of bracing or at surgery	44.2 (18.7) (14.0–77.0)	35.8 (19.4) (14.0–77.0)	51.9 (14.8) (29.0–77.0)	*p* = 0.0045
Completed treatment, i.e. end of bracing or after surgery	26.5 (13.1) (9.0–68.0)	22.6 (10.0) (9.0–41.0)	30.0 (14.8) (10.0–68.0)	ns (0.17)
First long-term FU, i.e. mean 26-y FU	36.6 (16.2) (9.0–83.0)	35.7 (17.8) (9.0–83.0)	37.5 (15.1) (14.0–73.0)	ns (0.54)
**Patients with curve size** **≥** **45° at**				
Start of treatment	18 (47.4%)	5 (27.8%)	13 (65.0%)	*p* = 0.047
Completed treatment/maturity	3 (7.9%)	0 (0.0%)	3 (15.0%)	ns (0.27)
`At the 26-Y follow-up	11 (28.2%)	5 (26.3%)	6 (30.0%)	ns (1.00)

*PF *Pulmonary function, *IS *Idiopathic scoliosis, *Early onset *Onset before age of 10 years

^a^Information regarding time for diagnoses was found in charts from the first visit, in some instances many years later. Especially for those with earlier diagnosis these figures might include some uncertainty. Ten patients had diagnosis before the age of six and twenty-nine from six to ten years of age

Significantly more patients in the reduced pulmonary function group had undergone surgery (70%) compared with the patients with normal pulmonary function (26%), also reflected by the findings that the R group was significantly younger at diagnosis of scoliosis (mean 5,2 years) and had larger curves at start of treatment (mean 52°) compared with the N group (mean 7,2 years, *p* = 0.018 and 36°, *p* = 0.0045, respectively).

Both groups had similar ages at the current follow-up, with mean ages of 47 years vs. 43 years for the R and N groups, respectively. The curve sizes at the 26 Y long-term FU were similar, 37° and 36°, respectively (n.s.). The time for follow-up from completed treatment until the 30-year long-term FU was 32 and 27 years, respectively.

Table 3 displays the results from spirometry and questionnaires.

**Table 3:** Pulmonary function (PF) and respiratory symptoms with change over time in early-onset idiopathic scoliosis

Mean (SD)/ (range) or *n*(%)	Total (*n* = 39)	NORMAL PF (*n* = 19)	REDUCED PF (*n* = 20)	*p*-value Normal vs. reduced
Total lung capacity (TLC) % of predicted				
26-Y long-term FU	84.0 (18.8) (38.5-109.7)	100.5 (4.5) (94.2-109.7)	68.3 (12.6) (38.5–80.2)	
30-Y long-term FU	86.6 (18.1) (46.7-126.2)	100.1 (5.2) (92.6-109.6)	73.7 (16.5) (46.7-126.2)	
Change from 1st to 2nd long-term FU	2.6 (13.4) (-9.1 to 64.3)	-0.3 (3.8) (-9.1 to 6.3)	5.3 (18.2) (-9.0 to 64.3)	ns (0.73)
**Forced vital capacity (FVC) % of predicted**				
26-Y long-term FU	81.4 (17.8) (36.0-109.9)	96.1 (7.1) (84.7-109.9)	67.5 (13.0) (36.0–93.0)	
30-Y long-term FU	79.5 (17.5) (41.3-109.8)	94.3 (6.6) (82.1-109.8)	65.4 (12.0) (41.3–85.6)	
Change from 1st to 2nd long-term FU	-2.0 (5.4) (-19.2 to 8.9)	-1.8 (3.9) (-9.3 to 7.9)	-2.1 (6.5) (-19.2 to 8.9)	ns (0.81)
**Forced expiratory volume in 1 s. (FEV1) % of predicted**				
26-Y long-term FU	75.9 (16.9) (30.8–108,2)	88.5 (8.7) (73.4-108.2)	64.1 (13.9) (30.8–89.5)	
30-Y long-term FU	74.5 (16.5) (36.9–111.1)	86.7 (9.7) (69.8–111.1)	62.9 (12.7) (36.9–84.2)	
Change from 1st to 2nd long-term FU	-1.5 (2.6) (-8.5 to 9.7)	1.7 (4.1) (-5.5 to 8.0)	1.2 (5.2) (-8.5 to 9.7)	ns (0.76)
**Respiratory symptoms**				
Dyspnea grading, occurrence of grade 4–5 a)				
26-Y long-term FU	3 (7.7%)	1 (5.3%)	2 (10.0%)	ns (1.00)
30-Y long-term FU	1 (2.6%)	0 (0.0%)	1 (5.0%)	
Wheezing, with or without infection				
26-Y long-term FU	13 (33.3%)	5 (26.3%)	8 (40.0%)	ns (0.57)
30-Y long-term FU	10 (25.6%)	5 (26.3%)	5 (25.0%)	
**Smoking habits**				
Smoker at present or ex-smoker	13 (33.3%)	8 (42.1%)	5 (25.0%)	ns (0.43)

*IS *Idiopathic scoliosis, *Early onset *Onset before age of 10 years

*PF *Pulmonary function was measured by TLC, FVC and FEV1. The results are presented as a percentage of the predicted values

Dyspnea severity shows individuals with grade 4 and 5, see Methods section

The decline in forced vital capacity % predicted between the 26 and 30 Y FUs was 2% in both groups, from a mean of 67 to 65% in the reduced function group and from 96 to 94% in the normal group during the 4-year follow-up (n.s.). TLC in % predicted showed no decline during the four-year follow-up.

The blood gas measurements of the 20 patients with reduced pulmonary function showed no signs of respiratory failure at the present follow-up. However, one patient showed reduced PaO_2_ (8,4 kPa). The forced vital capacity in this patient was 81% of the predicted normal, FEV1 77,5%, PaCO_2_ 5,0 kPa and standard bicarbonate 25. In one other patient, PaCO_2_ was 6,0, and FVC was 60% of the predicted normal. One further patient had an elevated SBC to 28. His FVC was 41% of the predicted normal.

There were no differences between the groups in terms of pulmonary symptoms, and no patient reported worsening dyspnea symptoms during the four-year follow-up, from the 26th until the 30th Y FU.

## Discussion

This study was performed to evaluate any further short-term deterioration in pulmonary function in patients with already reduced pulmonary function at the 26 year follow-up. Such a short-term deterioration could be regarded as a risk factor for later pulmonary failure [4]. The changes in FVC in per cent predicted over the four-year period did not differ between those with reduced and those with normal pulmonary function, although there was a marginal nominal decrease. In contrast, TLC showed a small nominal increase in the group with low pulmonary function.

We selected patients with the most severe pulmonary function deficit, identified by TLC (total lung capacity, as per cent of predicted) and compared with patients with the best pulmonary function from the same study group.

To our knowledge, the risk for a rapid decline in pulmonary function and ultimately respiratory failure in patients with severe idiopathic scoliosis treated with a brace or surgically treated has not been reported in the literature. Alveolar hypoventilation is regarded as the predominant mechanism for respiratory failure in severe scoliosis. The main risk for hypoventilation is during the night, as both the supine position and sleep may induce disturbed respiration [14]. The main measurement for detecting hypoventilation is regarded as an elevated PaCO_2_.

In this study, we found no patients with signs of respiratory failure and hypoventilation. Our measurements of arterial blood gases were, however, performed during the daytime. Therefore, we also evaluated standard bicarbonate values, reflecting long-standing elevation of PaCO_2_, which could be regarded as a good marker for night-time hypoventilation. Only one patient was found to have a somewhat higher value of the standard bicarbonate, although still within the normal range. In another patient, the oxygen tension was just below 10, which does not fulfill the definition of respiratory failure, and in no patient was the PaCO_2_ over 6,5 kPa. Thus, we could not detect any evident sign of hypoventilation in this study, with the possible exception of the only patient with an elevated standard bicarbonate.

However, we found three patients with FVC in per cent predicted lower than 50, a level where one cannot exclude the risk for developing respiratory failure later in life. One of these patients showed elevated SBC. Spirometric follow-up of patients with onset of scoliosis before the age of ten is therefore important. A level of FVC per cent predicted below 50 increases the indication for also performing arterial blood gas analysis.

Other factors, which have not been included in this study or in our earlier studies, might also influence the risk of developing respiratory problems for these patients in the long term.

A limitation of this study is that the relatively low number of patients as well as the relatively short follow up time might give a low power to detect a difference. However, as our earlier study showed that in untreated patients with diagnosis before the age of 10 the most severe risk of mortality occurred at an age over 40(1), we reasoned that an important risk in the present patients would show up in the current study.

## Conclusions

During this four-year follow-up of middle-aged patients with idiopathic scoliosis with onset before the age of ten and with reduced pulmonary function, we could not detect any difference in the rate of decline compared to patients with normal pulmonary function. Furthermore, we could not find any obvious signs indicating an increased risk for respiratory failure.

## Data Availability

The datasets used and/or analyzed during the current study are available from the corresponding author on reasonable request.

## Abbreviations

FU: Follow-up
FVC: Forced vital capacity
FEV1: Forced expiratory volume in one second
IS: Idiopathic scoliosis
N: Normal
PA: Posterior – anterior
PF: Pulmonary function
R: Reduced
SBC: Standard bicarbonate
SD: Standard deviation
TLC: Total lung capacity
Y: Year(s)
